# Supplementary material for: Branched flows of flexural elastic waves in non-uniform cylindrical shells
Source: PLoS One. 2023 May 26;18(5):e0286420. doi: 10.1371/journal.pone.0286420 (PMC10218726; doi:10.1371/journal.pone.0286420)
Supplement: S1 File — (PDF) [file pone.0286420.s001.pdf]

Supplementary Information: Branched flows of flexural elastic waves in non-uniform cylindrical shells

Kevin Jose and Neil Ferguson
Faculty of Engineering and Physical Sciences, University of Southampton, Southampton, SO17 1BJ, UK

Atul Bhaskar*
Department of Mechanical Engineering, University of Sheffield, Mappin Street, Sheffield, S1 3JD, UK

DERIVATION OF SCALING LAW FROM EQUATIONS OF MOTION OF CYLINDRICAL SHELLS

The equation of motion for the radial displacement of a cylindrical shell, $\eta(x,s,t)$, is given by (Yu [S1])

$$\frac{H^2}{12}\nabla^8\eta + \frac{1-\nu^2}{R^2}\partial_{xxxx}\eta + \rho\frac{2(1+\nu)}{E}\partial_{tt}\left[\left(\rho\frac{1-\nu^2}{E}\partial_{tt} - \frac{3-\nu}{2}\nabla^2\right)\right. \\ \left. \times\left(\rho\frac{1-\nu^2}{E}\partial_{tt}\eta + \frac{\eta}{R^2} + \frac{H^2}{12}\nabla^4\eta\right) + \frac{1-\nu}{2}\nabla^4\eta + \frac{\nu^2}{R^2}\partial_{xx}\eta + \frac{1}{R^2}\partial_{ss}\eta\right] = 0, \tag{S1}$$

where $\nabla^2 = \partial_{xx} + \partial_{ss}$. The material parameters, ν, E and ρ – Poisson's ratio, Young's Modulus and density respectively, are constant throughout.

This formalism ignores contributions to strain energy due to through thickness shear deformation, rotary inertia, and geometric non-linearity. The time independent terms in the resulting shell dynamics equations above are the same as Donnell's equations [S2] for statics.

Note that the system of equations given by Yu have expressions for the time evolution of the displacement in the circumferential and axial directions too. Equation S1 describes the time evolution of the radial displacement that is independent of the displacements in the other two directions. If we were interested in the displacements in the other two directions, they could be obtained in conjunction with the solution of Equation S1.

Eikonal equation and dispersion relations

We assume that $\lambda \ll L_c$. This means that $H(x,s)$ has properties that are spatially "slowly varying" which allows us to derive the eikonal equation from Equation S1. This is done by setting $\eta(x,s,t) = \hat{\eta}(x,s)e^{-i\omega t + iS(x,s)}$, where $S(x,s)$ is the eikonal. All derivatives of H and all derivatives of S higher than the first order are discarded due to the slowness of the spatial varitation of the properties. The eikonal equation thus obtained is

$$\frac{H^2(\partial_x S)^8}{12} + \frac{1}{3}H^2(\partial_x S)^6(\partial_s S)^2 + \frac{H^2(\partial_x S)^6\left(\nu^2-2\nu-3\right)\rho\omega^2}{12E} + \frac{1}{2}H^2(\partial_x S)^4(\partial_s S)^4 \\ + \frac{H^2(\partial_x S)^4(\partial_s S)^2\left(\nu^2-2\nu-3\right)\rho\omega^2}{4E} - \frac{(\partial_x S)^4\left(\nu^2-1\right)\left(H^2(\nu+1)\rho^2R^2\omega^4 - 6\rho R^2\omega^2E + 6E^2\right)}{6R^2E^2} \\ + \frac{1}{3}H^2(\partial_x S)^2(\partial_s S)^6 + \frac{H^2(\partial_x S)^2(\partial_s S)^4\left(\nu^2-2\nu-3\right)\rho\omega^2}{4E} - \frac{(\partial_x S)^2(\partial_s S)^2\left(\nu^2-1\right)\rho\omega^2\left(H^2(\nu+1)\rho\omega^2 - 6E\right)}{3E^2} \\ + \frac{H^2(\partial_s S)^8}{12} + \frac{H^2(\partial_s S)^6\left(\nu^2-2\nu-3\right)\rho\omega^2}{12E} - \frac{(\partial_s S)^4\left(\nu^2-1\right)\rho\omega^2\left(H^2(\nu+1)\rho\omega^2 - 6E\right)}{6E^2} \\ + \frac{(\partial_x S)^2\left(\nu^2-1\right)\rho\omega^2\left(\left(\nu^2-2\nu-3\right)\rho R^2\omega^2 + (2\nu+3)E\right)}{R^2E^2} \\ + \frac{(\partial_s S)^2\left(\nu^2-1\right)\rho\omega^2\left(\left(\nu^2-2\nu-3\right)\rho R^2\omega^2 + E\right)}{R^2E^2} \\ - \frac{2(\nu+1)\left(\nu^2-1\right)\rho^2\omega^4\left(\left(\nu^2-1\right)\rho R^2\omega^2 + E\right)}{R^2E^3} = 0. \tag{S2}$$

* a.bhaskar@sheffield.ac.uk

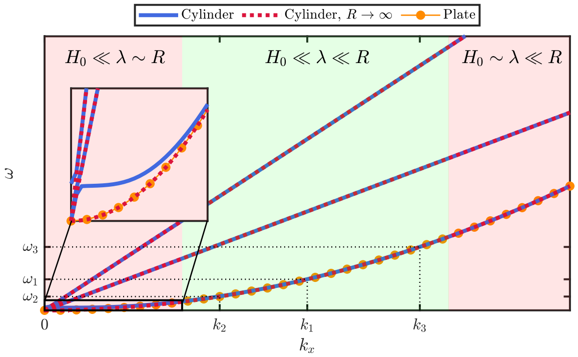

FIG. S1. **Comparison of dispersion relations** Dispersion relations obtained from equation of motion of a cylindrical shell are plotted with blue dotted lines; the same dispersion relation at the $R \to \infty$ limit is shown with orange dotted lines; the dispersion relation obtained from the equation of motion of a flat plate is plotted with a yellow dotted line. It can be seen that, in the parameter regime of interest ($H_0 \ll \lambda \ll R$), the lowest branch of the dispersion relations from equation of motion a cylinder is approximated well by the dispersion relation obtained from equation of a flat plate.

By setting $\partial_s S = 0,\ \partial_x S = k_x,\ H = H_0$, one gets the dispersion relation for flexural wave transmission along the axial direction in a uniform cylinder,

$$\begin{aligned} &\frac{k_x^4\left(H_0^2 k_x^4 R^2 - 12\nu^2 + 12\right)}{12R^2} \\ &+ \frac{k_x^2(\nu+1)\rho\left(H_0^2 k_x^4(\nu-3)R^2 + 12k_x^2(\nu-1)R^2 + 12\left(2\nu^2+\nu-3\right)\right)}{12R^2E}\omega^2 \\ &- \frac{(\nu-1)(\nu+1)^2\rho^2\left(H_0^2k_x^4R^2 - 6k_x^2(\nu-3)R^2 + 12\right)}{6R^2E^2}\omega^4 \\ &- \frac{2(\nu-1)^2(\nu+1)^3\rho^3}{E^3}\omega^6 = 0, \end{aligned} \tag{S3}$$

where k_x is the axial wavenumber. Further, setting $R \to \infty$ would yield the dispersion relation for a flat plate.

$$\begin{aligned} &\frac{H_0^2 k_x^8}{12} + \frac{k_x^4(\nu+1)\rho\left(H_0^2k_x^2(\nu-3) + 12(\nu-1)\right)}{12E}\omega^2 \\ &- \frac{k_x^2(\nu-1)(\nu+1)^2\rho^2\left(H_0^2k_x^2 - 6\nu + 18\right)}{6E^2}\omega^4 - \frac{2(\nu-1)^2(\nu+1)^3\rho^3}{E^3}\omega^6 = 0. \end{aligned} \tag{S4}$$

This can be compared with the dispersion relation for a flat plate [S3],

$$\omega^2 = \frac{EH_0^2k_x^4}{12\rho(1-\nu^2)}. \tag{S5}$$

We anticipate that all three dispersion relations will behave similarly in the regime of our interest ($H_0 \ll \lambda \ll R$). This can be seen from Figure S1 where the three dispersion relations have been plotted for typical material and geometric parameters used in the current study. We can see that in the parameter regime of interest, the lowest branch of the dispersion relations obtained from the equation of motion for a cylinder have similar values as those of the dispersion relation obtained from the equations of motion of a plate.

For the numerical study, we excite the elastic tubes by imposed displacements at a certain dominant frequency, shown in Figure S1 as $\omega_1, \omega_2, \omega_3$. The dispersion relations obtained from the equations of motions of a cylinder yield more branches as compared to the one from equations of a plate. This is because of the higher order of the governing equation and the other branches corresponding to other flexural and membrane-type displacements. However, it is seen from numerical simulations (FE) that the higher branches are not appreciably excited. This is because the excitation is predominantly radial. Hence, we can restrict ourselves to the lowest branch of the dispersion relation. As noted earlier, this lowest branch can be satisfactorily approximated by the dispersion relation obtained from equations of a plate in the parameter regimes of interest. Given the formal simplicity of the dispersion relation obtained from plate

equations we shall use them later, to write down k_x explicitly in terms of ω in the derivation of non-dimensionalised ray equations.

The red region on the left in Figure S1 corresponds to small wave-numbers or, alternatively, large wavelengths. When the wavelengths become comparable to the radius of the cylinder i.e. $\lambda \sim R$, the curvature plays an increasingly important role in the dynamics of wave propagation. This explains why the dispersion relations for a cylinder (with finite R) and dispersion relations in the $R \to \infty$ limit begin to diverge from each other in this region (see Figure S1, inset). Meanwhile, the red region on the right-hand side of Figure S1 corresponds to large wave-numbers or, alternatively small wavelengths (similar order as H_0). This is a regime ($H_0 \sim \lambda$) in which the suitability of the *thin* plate and shell equations we used to derive the dispersion relations is itself questionable.

Ray equations

By setting $\nabla S = [k_x \; k_s]^T$, where k_x & k_s are wavenumbers in the axial and circumferential directions respectively and then using Cauchy's method of characteristics (see [S3]), we obtain the ray equations

$$\partial_\tau x = \frac{k_x}{216R^2(1-\nu)}\Bigg[-H_0^6 k^8 R^2\left(k_x^2+k_s^2\right) + 3H_0^4 k_0^4(\nu-3)R^2\left(k_0^4 - 3\left(k_x^2+k_s^2\right)^2\right) \tag{S6}$$

$$- 36H_0^2(\nu-1)\left(k_0^4\left(2\nu + 2k_x^2R^2 + 2k_s^2R^2 + 3\right) - 4R^2\left(k_x^2+k_s^2\right)^3\right) - 864(\nu-1)^2(\nu+1)k_x^2\Bigg]$$

$$\partial_\tau s = \frac{H_0^2 k_s}{216R^2(1-\nu)}\Bigg[H_0^2 k_0^8 R^2\left(H_0^2\left(k_x^2+k_s^2\right) - 3\nu + 9\right) + 9k_0^4\Bigg(H_0^2(\nu-3)k_x^4R^2 + 2k_x^2R^2\left(H_0^2(\nu-3)k_s^2 + 4(\nu-1)\right)$$

$$+ H_0^2(\nu-3)k_s^4R^2 + 4(\nu-1) + 8(\nu-1)k_s^2R^2\Bigg) - 144(\nu-1)R^2\left(k_x^2+k_s^2\right)^3\Bigg]$$

$$\begin{bmatrix}\partial_\tau k_x \\ \partial_\tau k_s\end{bmatrix} = \frac{\nabla h}{432(1-\nu)}\Bigg[H_0^2\left(k_x^2+k_s^2\right)^2\left(H_0^2k_0^4 - 12\left(k_x^2+k_s^2\right)\right)\left(H_0^2k_0^4 + 6(\nu-1)\left(k_x^2+k_s^2\right)\right)\Bigg].$$

Here, k_0 is the wavenumber excited in the structure on excitation at frequency ω. Notice that the eikonal equation is only unique up to a multiplicative constant. Hence the scaling of the time variable τ is arbitrary, as is the case with ray approximations.

For the purpose of our discussions it would be useful to define two non-dimensional parameters which strongly influence the transmission of flexural waves in thin cylinder: (1) $\gamma = \frac{1}{k_0 R}$ and (2) $\epsilon = H_0 k_0$.

We will now non-dimensionalise the ray equations using $\tilde{x} = x/R$, $\tilde{s} = s/R$, $\tilde{k}_x = k_x/k_0$, $\tilde{k}_s = k_s/k_0$. We shall also replace τ with $\left(\frac{R}{k^7 H_0}\right)\tau$ as we can scale time arbitrarily. This scaling simplifies the non-dimensionalized ray equations. It also ensures that the time derivative terms are roughly $\mathcal{O}(1)$. This helps avoid any possible machine precision/overflow based issues when the ray equations are integrated numerically. The numerical integration worked fine without this scaling also. Machine precision/overflow issues might be rare for the system under consideration. However, we choose this scaling as a matter of good numerical computing practice. Apart from the two stated above, there are no other reasons to choose this specific scaling for τ. We shall use the expression $\omega = \sqrt{\frac{EH_0^2k_0^4}{12\rho(1-\nu^2)}}$ from dispersion relations of a flat plate. Ideally, we should obtain an expression for k_0 in terms of ω by solving the dispersion relation we obtained earlier for wave propagation in cylinders. While it may be possible to write it in closed form, it is expected to be algebraically complicated. Given that, we had earlier established, using observations from Figure S1, that the dispersion relation for a flat plat approximates the dispersion relation of a cylinder of the geometric and

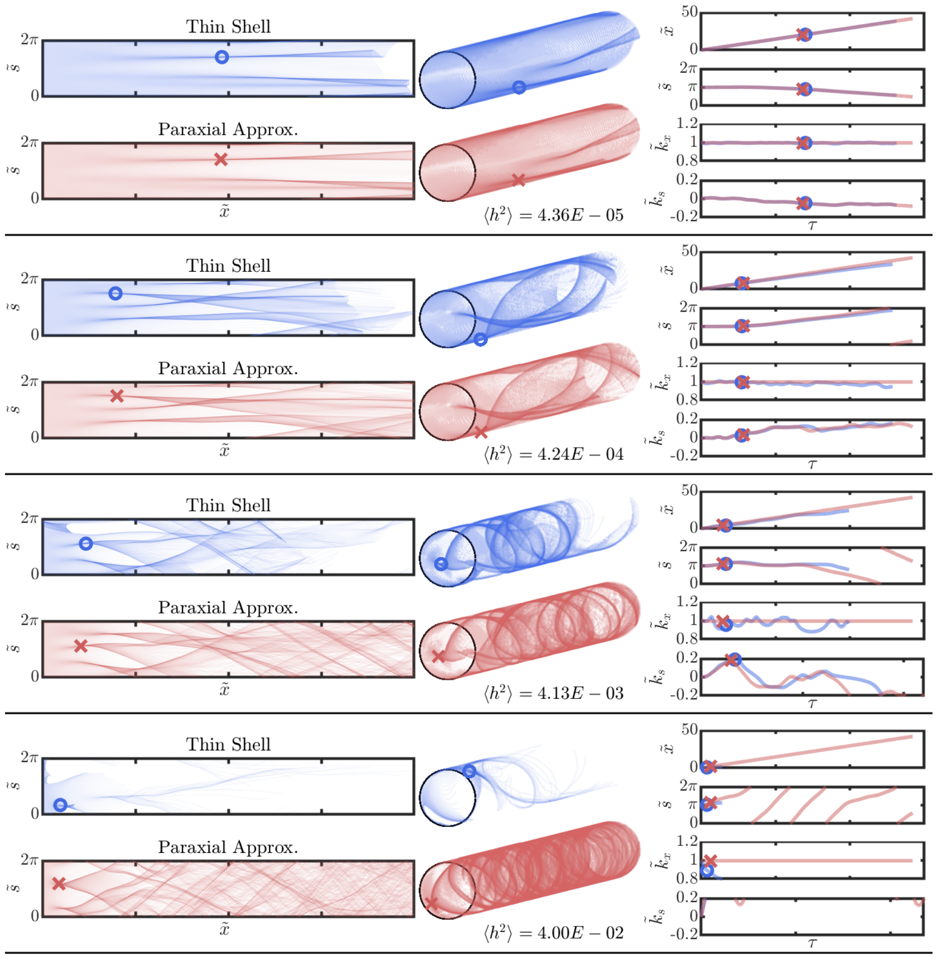

FIG. S2. **Comparison of thin shell ray equations and equations obtained from making the paraxial approximation using Yu's formulation.** Left: Ray propagation using full thin shell theory and paraxial approximation of thin shell theory. Circular and cross markers indicate location of the first caustic. The $\tilde{s}$ axis has been "unwrapped" for representational purposes. Middle: Same ray propagation shown on the cylindrical shell. Right: Plot of the temporal evolution of quantities describing the one of the rays. Here, markers indicate *temporal* location of the first caustic. It can be seen that, at higher values of $\langle h^2 \rangle$, complete backscattering of rays is observed in the thin shell case. This not modeled by the paraxial approximation which assumes weak scattering.

material parameters of interest,

$$\begin{aligned}
\partial_\tau \tilde{x} =& \frac{-\tilde{k}_x}{216(\nu-1)\epsilon^2}\Bigg[\tilde{k}_x^2\Bigg(18(\nu-3)\epsilon^4\tilde{k}_s^2 - 432(\nu-1)\epsilon^2\tilde{k}_s^4 + 864\gamma^2(\nu-1)^2(\nu+1) \\
&+ \epsilon^6 + 72(\nu-1)\epsilon^2\Bigg) + 9\tilde{k}_x^4\left((\nu-3)\epsilon^4 - 48(\nu-1)\epsilon^2\tilde{k}_s^2\right) - 144(\nu-1)\epsilon^2\tilde{k}_x^6 \\
&+ \epsilon^2\Bigg(-144(\nu-1)\tilde{k}_s^6 + \tilde{k}_s^2\left(72(\nu-1)+\epsilon^4\right) + 9(\nu-3)\epsilon^2\tilde{k}_s^4 \\
&+ 36\gamma^2\left(2\nu^2+\nu-3\right) - 3(\nu-3)\epsilon^2\Bigg)\Bigg], \\
\partial_\tau \tilde{s} =& \frac{-\tilde{k}_s}{216(\nu-1)}\Bigg[-144(\nu-1)\tilde{k}_x^6 + 9\tilde{k}_x^4\left((\nu-3)\epsilon^2 - 48(\nu-1)\tilde{k}_s^2\right) \\
&+ \tilde{k}_x^2\left(-432(\nu-1)\tilde{k}_s^4 + 18(\nu-3)\epsilon^2\tilde{k}_s^2 + 72\nu + \epsilon^4 - 72\right) - 144(\nu-1)\tilde{k}_s^6 \\
&+ \tilde{k}_s^2\left(72\nu+\epsilon^4-72\right) + 9(\nu-3)\epsilon^2\tilde{k}_s^4 + 36\gamma^2\nu - 36\gamma^2 - 3\nu\epsilon^2 + 9\epsilon^2\Bigg], \\
\begin{bmatrix}\partial_\tau \tilde{k}_x \\ \partial_\tau \tilde{k}_s\end{bmatrix} =& \frac{-\tilde{\nabla}\tilde{h}}{432(1-\nu)}\left(\tilde{k}_x^2+\tilde{k}_s^2\right)^2\left(12(\tilde{k}_x^2+\tilde{k}_s^2)-\epsilon^2\right)\left(6(\nu-1)(\tilde{k}_x^2+\tilde{k}_s^2)+\epsilon^2\right),
\end{aligned} \tag{S7}$$

where $\tilde{\nabla} = [\partial_{\tilde{x}}\ \partial_{\tilde{s}}]^T$.

The ray equations derived from the equations of motion of transverse displacement of a cylindrical shells are rather complicated and not particularly amenable to further analysis. However, in the regime of weak scattering that we study here, the ray equations can be simplified greatly. $\tilde{k}_x$ and $\tilde{k}_s$, the wavenumbers in the x and s directions are not expected to vary substantially from their initial values of 1 and 0 respectively, as the initially launched plane wave is purely axial. For weak scattering of initially plane waves, it is customary to make the simplifying assumption that the wave-number does not change at all in the main propagation direction. This can be achieved by setting $\partial_\tau \tilde{k}_x = 0$, $\tilde{k}_x = 1$ in Equation S7, which is the essence of the paraxial approximation. Finally, dropping all $\tilde{k}_s$ terms compared to $\mathcal{O}(1)$ terms, the ray equations become

$$\partial_\tau \tilde{x} = c_1, \qquad \partial_\tau \tilde{s} = c_2\tilde{k}_s, \qquad \partial_\tau \tilde{k}_x = 0, \qquad \partial_\tau \tilde{k}_s = c_3\tilde{h}_{\tilde{s}}, \tag{S8}$$

where, $c_1 = \gamma^2(-\nu/3 + (1-\nu^2)4/\epsilon^2 - 1/2) + \zeta$, $c_2 = -\gamma^2/6 + \zeta$, $c_3 = \zeta/2$, $\zeta = -\frac{(\epsilon^2-12)(6\nu+\epsilon^2-6)}{216(\nu-1)}$.

Figure S2 shows the comparison of ray equations from thin shell theory and the one obtained after applying the paraxial approximation. The figure is similar to Fig. 2 in the main article. However, here we can see that the ray equations based on thin shell theory are able to model even complete back scattering at higher values of $\langle h^2\rangle$ unlike the formulations of ray equations used in the main text. Nonetheless, it can be seen that after the application of the paraxial approximation (which negates any back-scattering behavior), the rays look identical to the one in the main text even at higher $\langle h^2\rangle$.

Figure S3 shows the scaling of $\langle l_f\rangle$ with $\langle h^2\rangle$ from all the sets of ray equations used in this work. Thin shell ray equations from Yu's formulation is the most accurate and is able to capture back scattering, that is expected at higher $\langle h^2\rangle$. This explains why thin shell equations from Yu's formulation show significant departure from the predicted exponential scaling at higher $\langle h^2\rangle$.

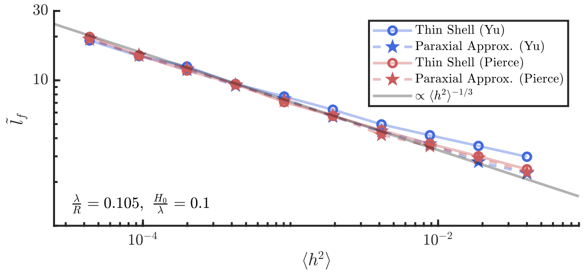

FIG. S3. **Comparison of $\langle l_f \rangle$ obtained from different ray equations**. $\langle l_f \rangle$ obtained from thin shell and paraxial ray equations from Pierce's and Yu's formulations. Thin shell equations from Yu's formulations shows significant deviation from the expected scaling at higher $\langle h^2 \rangle$, as expected.

Scaling law from the analysis of ray equations

We use the ray equations obtained after making the paraxial approximations Equation S8, and following a process very similar to other branched flow works [S4, S5] we obtain, $\langle l_f \rangle \propto \alpha(\gamma, \epsilon) L_c \langle h^2 \rangle^{-1/3}$ where

$$\alpha(\gamma, \epsilon) = \left(\frac{3}{2\sqrt{\pi}} \frac{\left(\gamma^2(-\nu/3 + (1-\nu^2)4/\epsilon^2 - 1/2) + \zeta\right)^4}{\left(-\gamma^2/6 + \zeta\right)^2 \left(\zeta/2\right)^2} \right)^{\frac{1}{3}}. \tag{S9}$$

Note that, α has an explicit dependence on γ & ϵ i.e. H_0, R & λ. However, it can be seen from Figure S4 that the dependence is actually quite weak in the parameter ranges of interest ($\alpha \approx 1.55 \pm 5\%$). Green cross markers indicate the cases that have been reported here. Therefore, we can conclude that the expected location of the first caustic is independent of wavelength, assuming the thickness is small, and the radius is large.

Numerical simulations using ray equations derived from Yu's formulation

In Figure S5, it can be seen that the scaling of $\langle l_f \rangle$ predicted in the main text is confirmed from numerical integration of the paraxial ray equations resulting from Yu's formulation of the equations of motion of a cylindrical shell. Details of other parameters used in numerical ray integration and FE simulations, reported here and in the main text, is in Table S1. It must be noted that our results are not dependent on this specific choice of parameter values, as confirmed by the analysis presented.

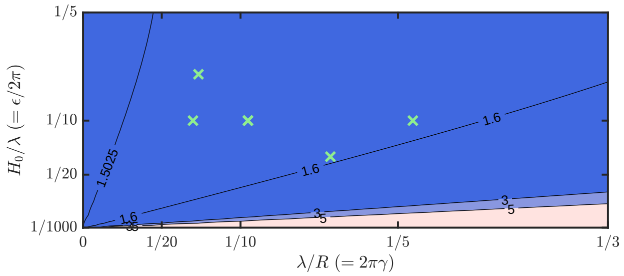

FIG. S4. **Plot of $\alpha(\gamma, \epsilon)$**. This plot shows that in the parameter region of interest, α has a weak dependence on γ & ϵ. Green cross markers indicate the parameters used in studies reported here.

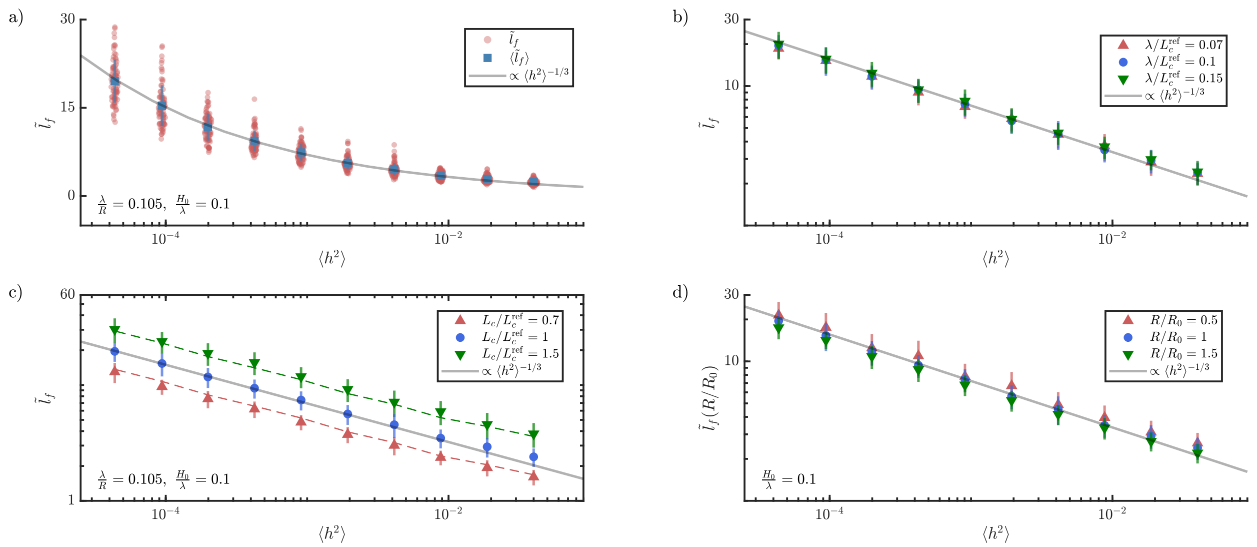

FIG. S5. **Scaling of $\langle l_f \rangle$ obtained from numerical ray integration of ray equations obtained from Yu's equations of motion.** a) Scaling of $\langle l_f \rangle$ with $\langle h^2 \rangle$ agrees well with the predicted exponential scaling. b) $\langle l_f \rangle$ is independent of wavelength as predicted. c) The linear scaling of $\langle l_f \rangle$ with L_c is confirmed. d) $\langle l_f \rangle$ does not scale with R in the parameter regime of interest.

Variable Name	Symbol	Units	Value(s)
Young's Modulus	E	MPa	**200**
Density	ρ	$\mathrm{kg\,m^{-3}}$	**7800**
Poisson's Ratio	ν	1	**0.3**
Correlation length	L_c	m	**0.1**, 0.07, 0.15
Radius	R	m	**0.0955**, 0.0477, 0.1432
Wavelength	λ	m	**0.01**, 0.007, 0.015
Nominal thickness	H_0	m	**0.001**
Length in propagation direction	L_x	m	4, 8 -depending on $\langle h^2 \rangle$

TABLE S1. **List of parameters used in numerical ray integration and FE simulations for all the results.** When it is unclear from the text, it may be assumed that the parameter values in bold face were used.

SUPPLEMENTARY REFERENCES

[S1] Y.-Y. Yu, Free Vibrations of Thin Cylindrical Shells Having Finite Lengths With Freely Supported and Clamped Edges, Journal of Applied Mechanics **22**, 547 (1955), https://doi.org/10.1115/1.4011152.

[S2] L. H. Donnell, *Stability of thin-walled tubes under torsion*, Tech. Rep. (National Advisory Committee for Aeronautics/NASA, 1935) https://ntrs.nasa.gov/citations/19930091553.

[S3] A. D. Pierce, Physical Interpretation of the WKB or Eikonal Approximation for Waves and Vibrations in Inhomogeneous Beams and Plates, The Journal of the Acoustical Society of America **48**, 275 (1970).

[S4] H.-P. Degueldre, *Random Focusing of Tsunami Waves*, Ph.D. thesis, Georg-August-Universitat Gottingen (2015), http://dx.doi.org/10.53846/goediss-5365.

[S5] J. J. Metzger, *Branched Flow and Caustics in Two-Dimensional Random Potentials and Magnetic Fields*, Ph.D. thesis, Georg-August-University Goettingen (2010), http://dx.doi.org/10.53846/goediss-2917.
