# Supplementary material for: Branched flows of flexural elastic waves in non-uniform cylindrical shells
Source: PLoS One. 2023 May 26;18(5):e0286420. doi: 10.1371/journal.pone.0286420 (PMC10218726; doi:10.1371/journal.pone.0286420)
Supplement: Attachment — Submitted filename: Plos-One Report.pdf [file pone.0286420.s003.pdf]

In this article, the authors investigate the propagation of elastic waves along the axis of cylindrical shells with geometric imperfections and spatial variations in properties. They report the existence of branched flows of flexural waves in such waveguides and derive the scaling laws from the ray equations. They showed that the numerical integration of the ray equations and finite element numerical simulations are consistent with the theoretically derived scaling. They also note a universality for the exponents in the scaling with respect to similar observations in the past for waves in other physical contexts, as well as dispersive flexural waves in elastic plates. The authors suggest an immediate extension of this work on cylindrical elastic shells to explore the dependence of the scaling of the first caustic with the radius for shells with appreciable curvature. However, they are unable to do so in this work due to the limitation of $2\pi R \gtrsim Lc$. They suggest using anisotropic randomness to enable reducing the radius by using a smaller correlation length in the circumferential direction and exploring an elegant scaling of $\langle lf \rangle$ with radius in this parameter regime.

Overall, this article presents a well-structured and well-executed study on the propagation of elastic waves in cylindrical shells with random spatial variations. The authors' findings are significant and contribute to the current understanding of branched flows in wave propagation through random media. The article provides theoretical, numerical, and analytical evidence to support the authors' claims and suggests future directions for research, and may be accepted for the publication once it is comprehended to answers the following question.

1) What are the practical applications of understanding the branched flows of flexural waves in cylindrical shells, and how can this knowledge be utilized in real-world scenarios?
2) Can the findings of this study be generalized to other types of structures or materials, or is it specific to cylindrical shells with correlated random properties?
3) What are the limitations of the numerical integration of the ray equations and the full FE elastodynamic simulations, and how accurate are these methods in predicting the behavior of elastic waves in cylindrical shells?
4) What are the potential implications of using anisotropic randomness to reduce the radius of cylindrical shells, and how would this affect the scaling of $\langle lf \rangle$ with radius in this parameter regime?
5) How can the results of this study be applied to improve the design and engineering of cylindrical shell structures, and what further research is needed to fully understand the behavior of elastic waves in such structures?

Some minor changes are:

1) On page 2, line 88, Eq (3) should be Eq (2).
2) On page 4, line 94 (details in Methods) can be deleted, and likewise details provided in rest of the article with figures and sentences.
