# Supplementary material for: Branched flows of flexural elastic waves in non-uniform cylindrical shells
Source: PLoS One. 2023 May 26;18(5):e0286420. doi: 10.1371/journal.pone.0286420 (PMC10218726; doi:10.1371/journal.pone.0286420)
Supplement: Attachment — Submitted filename: Rebuttal Letter - Response to reviewers.pdf [file pone.0286420.s004.pdf]

Dear Editor and Reviewers,

We have revised our manuscript based on the comments and suggestions by the reviewers. Our responses (in bold blue font) to reviewer comments are appended below.

We thank the reviewers for their comments and suggestions. They have, undoubtedly, improved this manuscript.

Reviewers' comments:

Reviewer's Responses to Questions

Comments to the Author

1. Is the manuscript technically sound, and do the data support the conclusions?

The manuscript must describe a technically sound piece of scientific research with data that supports the conclusions. Experiments must have been conducted rigorously, with appropriate controls, replication, and sample sizes. The conclusions must be drawn appropriately based on the data presented.

Reviewer #1: Yes

Reviewer #2: Yes

---

2. Has the statistical analysis been performed appropriately and rigorously?

Reviewer #1: Yes

Reviewer #2: Yes

---

3. Have the authors made all data underlying the findings in their manuscript fully available?

The PLOS Data policy requires authors to make all data underlying the findings described in their manuscript fully available without restriction, with rare exception (please refer to the Data Availability Statement in the manuscript PDF file). The data should be provided as part of the

manuscript or its supporting information, or deposited to a public repository. For example, in addition to summary statistics, the data points behind means, medians and variance measures should be available. If there are restrictions on publicly sharing data—e.g. participant privacy or use of data from a third party—those must be specified.

Reviewer #1: Yes

Reviewer #2: Yes

---

4. Is the manuscript presented in an intelligible fashion and written in standard English?

PLOS ONE does not copyedit accepted manuscripts, so the language in submitted articles must be clear, correct, and unambiguous. Any typographical or grammatical errors should be corrected at revision, so please note any specific errors here.

Reviewer #1: Yes

Reviewer #2: Yes

---

5. Review Comments to the Author

Please use the space provided to explain your answers to the questions above. You may also include additional comments for the author, including concerns about dual publication, research ethics, or publication ethics. (Please upload your review as an attachment if it exceeds 20,000 characters)

Reviewer #1: The manuscript is concerned with branched flows of flexural waves in non-uniform elastic cylindrical shells with slow spatial variation of properties. The scaling laws involving the expected distance between the point of launch and the first of the focusing events, the correlation length of the isotropic randomness field, and a non-dimensional measure of the degree of randomness, are derived theoretically from the ray equations. The ray equations are solved numerically to illustrate the emergence of branched flow and verify the theoretical prediction for the scaling law. Finite element simulations are also performed to present the full wave elastodynamics.

The manuscript is within the scope and of clear interest for the journal. This is a challenging but useful problem. I am happy to suggest its acceptance, after the authors address the comments

listed below.
1. The state of art could be slightly better described. In particular, for delicate aspects of applying WKB method for thin shells see Kaplunov and Tovstik (Localized Dynamics of Thin-Walled Shells, 2020) and references therein; for classification of dynamic regimes in thin elastic shells and limitations of the theory see Kaplunov, Kossovich and Nolde (Dynamics of Thin Walled Elastic Bodies, 1998).

We have expanded the section title "Introduction" based on this suggestion. We have also added four new references (the two suggested by the referee, and another two related to localised wave motion – such as Raleigh surface waves in an elastic half-space and Konenkov waves at the edge of elastic plates).

2. It would be good to explain about the first caustics in slightly more detail, possibly with more formulae?

Based on this suggestion, we have expanded the sub-section titled "Detecting first focusing events" to provide a more mathematical description of what constitutes a caustic.

3. Since the authors have previously studied branched flows of flexural elastic waves in plates [7], it would be good to compare what are the novel features emerging in shells?

Based on this suggestion, we have added a sentence describing the emergence of spiral waves in branched flow in cylindrical shells which are absent in plates due to reflecting boundaries.

4. In formulae (2) in brackets what is the purpose of putting numerators of the fraction in brackets, could we have it without, say, \tilde{k}_x^4 in case of the second equation? In this case in the first equation the sign minus from the numerator could appear in front of the fraction?

We have made this suggested change in the manuscript.

5. Please reformulate the sentences start with a formula, e.g. "τ is an arbitrary time variable, consistent with ray approximations" (page 3, line 78), could become "Here τ…".

We have made changes to the manuscript based on this suggestion

Reviewer #2: In this article, the authors investigate the propagation of elastic waves along the axis of cylindrical shells with geometric imperfections and spatial variations in properties. They report the existence of branched flows of flexural waves in such waveguides and derive the scaling laws from the ray equations. They showed that the numerical integration of the ray equations and finite element numerical simulations are consistent with the theoretically derived scaling. They also note a universality for the exponents in the scaling with respect to similar observations in the past for waves in other physical contexts, as well as dispersive flexural waves in elastic plates. The authors suggest an immediate extension of this work on cylindrical elastic shells to explore the dependence of the scaling of the first caustic with the radius for shells with appreciable curvature. However, they are unable to do so in this work due to the limitation of $2\pi R \gtrsim Lc$. They suggest using anisotropic randomness to enable reducing the radius by using a smaller correlation length in the circumferential direction and exploring an elegant scaling of $\langle lf \rangle$ with radius in this parameter regime.
Overall, this article presents a well-structured and well-executed study on the propagation of elastic waves in cylindrical shells with random spatial variations. The authors' findings are significant and contribute to the current understanding of branched flows in wave propagation through random media. The article provides theoretical, numerical, and analytical evidence to support the authors' claims and suggests future directions for research, and may be accepted for the publication once it is comprehended to answers the following question.
1) What are the practical applications of understanding the branched flows of flexural waves in cylindrical shells, and how can this knowledge be utilized in real-world scenarios?

We have made changes to the manuscript based on this suggestion and added a short discussion of potential application to metrology in the section titled "Conclusions"

2) Can the findings of this study be generalized to other types of structures or materials, or is it specific to cylindrical shells with correlated random properties?

We have added a new section titled "Discussions", where this query has been addressed.

3) What are the limitations of the numerical integration of the ray equations and the full FE elastodynamic simulations, and how accurate are these methods in predicting the behavior of elastic waves in cylindrical shells?

We have added a new section titled "Discussions", where this query has been addressed.

4) What are the potential implications of using anisotropic randomness to reduce the radius of cylindrical shells, and how would this affect the scaling of $\langle lf \rangle$ with radius in this parameter regime?

We have added a new section titled "Discussions", where this query has been addressed.

5) How can the results of this study be applied to improve the design and engineering of cylindrical shell structures, and what further research is needed to fully understand the behavior of elastic waves in such structures?

We have added a new section titled "Discussions", where this query has been addressed.

Some minor changes are:
1) On page 2, line 88, Eq (3) should be Eq (2).

We have made changes to the manuscript based on this suggestion

2) On page 4, line 94 (details in Methods) can be deleted, and likewise details provided in rest of the article with figures and sentences.

We have made changes to the manuscript based on this suggestion

---

6. PLOS authors have the option to publish the peer review history of their article (what does this mean?). If published, this will include your full peer review and any attached files.

If you choose "no", your identity will remain anonymous but your review may still be made public.

Do you want your identity to be public for this peer review? For information about this choice, including consent withdrawal, please see our Privacy Policy.

Reviewer #1: No

Reviewer #2: No

Yours sincerely,
Atul Bhaskar
Professor of Applied Mechanics
Department of Mechanical Engineering
University of Sheffield
